# An Investigation Into the Interplay Between Chinese EFL Teachers' Emotional Intelligence, Ambiguity Tolerance, and Work Engagement

**DOI:** 10.3389/fpsyg.2022.929933

**Published:** 2022-07-13

**Authors:** Nan Yang

**Affiliations:** School of Humanity, Shandong Management University, Jinan, China

**Keywords:** ambiguity tolerance, emotional intelligence, work engagement, EFL teachers, predictability power

## Abstract

Teachers' work engagement is regarded as a critical issue in educational contexts, so the emotional factors and personality traits, and their effects on teacher engagement have drawn the attention of investigators. This study seeks to investigate the relationship between teachers' emotional intelligence, ambiguity tolerance, and work engagement. Moreover, this study tries to investigate the contribution of emotional intelligence and ambiguity tolerance to teachers' work engagement. To do so, 322 teachers (96 males and 226 females) participated in this study. Schutte's Self Report Emotional Intelligence Test (SSEIT), Multiple Stimulus Types Ambiguity Tolerance Scale-II (MSTAT-II), and Self-report engagement Questionnaire were used in this study. The statistical techniques used in this study are the Spearman Rho test and ANOVA. The findings showed that there are significant correlations between work engagement, emotional intelligence, and ambiguity tolerance. Comparing the predictability power, teachers' emotional intelligence (*B* = 0.611) proved to have a higher index compared to their index of ambiguity tolerance (*B* = 0.2). This study concluded that emotionally intelligent teachers and teachers with higher levels of ambiguity tolerance are more engaged in the EFL contexts. Moreover, the study has some pedagogical implications and suggestions for different teacher educators, policy-makers, and advisors. The ideas can improve their awareness of teachers' emotional intelligence, ambiguity tolerance, and work engagement in educational environments.

## Introduction

Emotional intelligence, as a psychological concept, is the result of the entanglement of both emotional and intellectual minds. Emotional intelligence is the relationship between reason and emotion, and since humans are often neither fully rational nor emotional, a person's ability to adapt to the environment and cope with life's problems depends on the combined function of emotional and intellectual abilities. Today, emotional intelligence has been the subject of much research on the study of individual differences. The ability to predict life success and the essential role of this structure in most mental disorders can be the reasons for the interest in studying emotional intelligence (Kurniawan and Syakur, 2017). Emotional intelligence theorists believe that there is a positive relationship between emotional intelligence, and the ability to cope with risky and ambiguous situations, and they have raised the level of emotional intelligence as a protective factor (Salovey et al., 1999). Many researchers take emotional intelligence as an important indicator in occupational and professional fields of education. Emotional intelligence leads to valuable life if the teacher knows how to take advantage of this skill. For academic excellence, teachers need to understand the difference between cognitive and emotional intelligence but they must focus on the emotional literacy of their students, and teachers' emotional literacy will show when teachers check their own emotional literacy (Zeidner et al., 2011; Habeb Al-Obaydi et al., 2022). Emotionally intelligent teachers show care for students, create an emotional climate in the classroom that develops the student learning environment and helps the teachers to become more effective to ensure an academic achievement. It has been seen that teacher's emotional intelligence affects their comfort level, self-efficacy, job satisfaction level and enhances their social relationship with students. As a result, emotional intelligence directly affects the teaching and learning process (Jennings and Greenberg, 2009). Working on classroom emotions has become vital nowadays for students' emotional positive growth or for positive academic achievement. It is hoped that successful teachers have a high level of emotional competencies. Emotional intelligence forecasts positive and successful results in all fields of life and consequently, it dominates all fields of education. Teachers need to be trained in emotional intelligence to manage their own emotions for helping students. This makes emotional intelligence has become important for both teachers and students (Singh, 2015).

Furthermore, Bisini and Musthafa (2015) also believe that individuals' tolerance of ambiguity affects their principles. Chapelle and Roberts (1986) defined tolerance of ambiguity as “a person's ability to function rationally and calmly in a situation in which interpretation of all stimuli is not clear” (p. 30). Monrouxe and Mattick (2006) also stated that regulating ambiguity is a critical strategy for job advancement. McLain (2009) highlighted the effect of ambiguity tolerance on individuals' insights and efficiency. Educational contexts are surrounded by difficulties and ambiguities. These ambiguities stem from numerous causes, including ambiguities in instructional approach, teaching materials, and students' learning processes (Berlak and Berlak, 1981).

Moreover, the concept of teachers' work engagement, in educational contexts, is significant but disregarded in the conventionalized EFL classroom contexts (Zhao et al., 2021). According to Schaufeli et al. (2002), teachers' work engagement refers to “a positive, fulfilling, work-related state of mind that is characterized by vigor, dedication, and absorption” (p. 202). They argued that dedicated and absorbed instructors can provide inspiring educational contexts in which learners tend to engage in the learning process. Teachers' work engagement can predict their teaching effectiveness, activities, problem-solving, and job satisfaction (Minghui et al., 2018). These variables show that educators' features are worth investigating to enhance their teaching activities. Conventionally, EFL educators' traits have been estimated based on their foreign language knowledge, qualifications, and experience. Nevertheless, studies have also recognized the significance of educators' approaches, viewpoints, and principles regarding their instruction to expedite learners' academic achievement (Ekstam et al., 2017).

Concepts such as emotional intelligence, ambiguity tolerance, and work engagement were considered to be significant variables in improving the performances of the teachers in the literature. However, there have been few previous investigations of teachers' work engagement which make it necessary for investigators to do research in this field. Having an awareness of teachers' emotional intelligence and ambiguity tolerance, and their relationship with work engagement can endorse and expand the positive psychological constructs. Moreover, by knowing about these variables, school managers would be able to make their teachers enthusiastic and improve their level of engagement. Furthermore, the investigation of psychological constructs, like teachers' emotional intelligence, ambiguity tolerance, and their work engagement can shed new light on the instruction.

### Research Questions

To this end, this study tries to answer the following questions:

Q1: What are the relationships between Chinese EFL teachers' work engagement, emotional intelligence, and ambiguity tolerance?
Q2: To what extent can EFL teachers' emotional intelligence and ambiguity tolerance predict teacher engagement in the Chinese EFL educational context?

## Literature Review

### The Notion of Ambiguity Tolerance

Brugnach and Ingram (2012) regarded ambiguity as “unrecognized contextual, methodological and substantive differences among knowledge systems” (p. 61). They maintained that a knowledge system denotes information, procedure, skills, practices, and principles that are established in a society, and applied as a starting point for judging. They also asserted that individuals require cohesive systematic knowledge in order to cope with ambiguity. Arquero et al. (2017) asserted that the ambiguity of an individual in unfamiliar situations leads to the restriction in decision-making and prediction. Furnham and Marks (2013) also pointed out that “ambiguity tolerance affects several facets of human's cognitive style, belief, value, and attitude systems, interpersonal skills, and problem-solving” (p. 22). Ambiguity tolerance “generalizes to the various aspects of emotional and cognitive functioning of the individual, characterizing cognitive style, belief and attitude systems, interpersonal and social functioning and problem-solving behavior” (Furnham and Marks, 2013, p. 717). Kornilova and Kornilov (2010) suggested distinguishing between tolerance for uncertainty and intolerance for uncertainty, defining the former as “readiness to make decisions and act in uncertain situations, openness to new ideas, changing stimuli and changing thinking strategies” (p.20). The latter was interpreted as “willingness to achieve clarity in the world (including the world of ideas), rejection of uncertainty in judgments, rigidity and rationality (as directed toward acquiring maximum information required for making a decision)” (p.21). It should be noted that their interpretation of tolerance for uncertainty is similar to that of ambiguity tolerance since the key aspects of the two constructs deals with openness, novelty, change and taking risk.

Furthermore, numerous investigators have found that ambiguity tolerance may be regarded as one of the most important features applied in the definition of an individual's personality (Li and He, 2016). Kazamia (1999), for example, argued that ambiguity tolerance is an aspect correlated with individuals' personality and their cognitive styles. Hadley (2003) also mentioned that tolerant individuals deal with complicated circumstances and accept them without getting irritated.

In educational contexts, many investigations have been done on learners' ambiguity tolerance (e.g., Seidi, 2018; Soodmand Afshar and Khasemy, 2019; Yu et al., 2021). Kamran and Maftoon (2012) believed that a good language learner is someone “who is often not inhibited and who is willing to make mistakes in order to learn and to communicate, and who is willing to live with a certain amount of vagueness (p. 188). Erten and Topkaya (2009) indicated that tolerant learners are inclined to use guessing strategies in educational environments. Moreover, they found that tolerant learners tend to use compensation strategies more than intolerant learners. Furthermore, Ashouri and Fotovatnia (2010) regarded linguistic inputs and cultural knowledge as causes of ambiguities. They argued that inadequate or lack of information about vocabularies or grammatical structures is learners' causes of ambiguity. Varasteh et al. (2016) found that learners' ambiguity tolerance influences learners' academic success in numerous tests and language skills. Moreover, Piechurska-Kuciel (2018) asserted that ambiguity tolerance affects learners' willingness to communicate in educational contexts. Atamanova and Bogomaz (2014) also indicated that EFL learners' ambiguity tolerance specifies learners' communicative competence. Regarding negative emotions, Dewaele and Ip (2013) stated that learners' ambiguity tolerance is negatively correlated with their anxiety and language proficiency. They argued that anxiety and ambiguity tolerance are extensions of “neuroticism” and “openness”. They also mentioned that anxious learners are less proficient and intolerant in ambiguous situations. They also mentioned that language proficiency increases learners' ambiguous tolerance.

On the other hand, Kamran (2011) indicated that ambiguity tolerance can facilitate and hinder language instruction depending on one's capability to cope with it. Few investigations have been done on teachers' ambiguity tolerance. Ambiguity tolerance has been correlated positively with innovativeness (Nicotera et al., 1990), a constructivist teaching orientation (Rittschof, 2016), and teachers' creativity (Tegano, 1990). Some studies have also been done on the relationship between teachers' personality types and teachers' personality types. Rezaei et al. (2019) argued that individuals, with higher levels of tolerance in ambiguous situations, do not take risk in their job. The literature also demonstrates that ambiguity tolerance is correlated with EFL instructors' negative emotions such as burnout (Zhaleh et al., 2018).

### The Notion of Emotional Intelligence

Emotional intelligence is defined as the awareness of an individual of his emotions and others' emotions and the ability to recognize and control them and also the ability to express sympathy for others. EQ deals with evaluating aspects of a situation (positive or negative) and making suitable solutions in stressful situations (Mayer et al., 2004). Accordingly, EQ is the ability to recognize emotions, to access and generate them in order to aid thought, comprehend emotions and emotional knowledge, and reflectively control them to advance emotional and intellectual growth (Mayer and Salovey, 1997). Ebrahimi et al. (2018) described emotional intelligence as a trait in individuals which empowers them to regulate their feelings and emotional states of others, distinguish between diverse feelings, and to apply emotional information to lead their thoughts and performances. Kliueva and Tsagari (2018) also stated that individuals with higher emotional intelligence are able to improve their interpersonal behaviors through developing intelligence, empathy, and feelings. Moreover, some personality features, including self-assurance, conscientiousness, and motivation for success were incorporated into the definition (Saud, 2019). Cherniss (2010) stated that emotional intelligence is concerned with the perception and regulation of emotions, whereas social competence relates to individuals' propensity to have emotional intelligence. In a nutshell, Wicks et al. (2018) asserted that emotional intelligence is regarded as an individuals' aptitude, ability, capability, and personality trait.

Some investigations have been done on learners' emotional intelligence and its relationship with their academic achievement (e.g., Ahmed et al., 2019; Karimi et al., 2020), language learning strategies (Zafari and Biria, 2014; e.g., Shabani and Ghodrati, 2018), willingness to communicate (e.g., Dastgoshadeh and Javanmardi, 2021; Taherkhani and Moradi, 2022) in educational contexts. Mortiboys (2013) asserted that language instruction is restricted to the knowledge of teaching and theories of learning. However, he mentioned that emotional intelligence, can be considered as an important component of instruction. The fundamental function of emotional intelligence, as a useful quality for the efficiency of instruction, has been supported in related studies (Khani and Ghasemi, 2019). Regarding teaching effectiveness, Chen and Guo (2020) asserted that emotional intelligence is a must for improving instructional effectiveness. It can improve leadership efficiency among teachers to foster their performance.

Some studies have been done on the relationship between teachers' emotional intelligence and their positive and negative emotions. Regarding negative emotions, Esmaili et al. (2018) found that three features of teacher burnout, including emotional exhaustion, depersonalization, and personal accomplishment are significantly correlated with their emotional intelligence. Regarding positive emotions, Barłozek (2015) examined instructors' emotional intelligence and its relationship with teacher rapport as a positive emotional construct. He stated that “teachers with higher levels of emotional intelligence were better assessed and perceived by the learners” (p.20). Dewaele et al. (2018), in a study on the relationship between emotional intelligence and the teacher-student relationship as a positive construct, revealed that EFL/ESL instructors' emotional intelligence and their rapport with learners are significantly correlated with each other. In another study, Puertas Molero et al. (2019) stated that teachers' emotional intelligence is a significant feature in educational contexts, which enables them to increase their wellbeing, which, in turn, fosters instructional methodologies. They argued that teachers' emotional intelligence increases their aptitude to control feelings, enhances their decision-making in the instructional contexts, along with increasing learners' academic achievement. Ngui and Lay (2020) also found out that teachers' emotional intelligence significantly predicts their resilience as a construct of positive psychology. Kostić-Bobanović (2020) also indicated that teacher' emotional intelligence is significantly correlated with their self-efficacy. He mentioned that the components of emotional intelligence including self-awareness, interpersonal relation, and problem-solving are significantly correlated with self-efficacy.

### Teachers' Work Engagement

A number of definitions of work engagement are available in the literature. Kahn (1990, p. 694) was the first researcher to define engagement, which he described as “the harnessing of organization member's selves to their work roles, and express themselves physically, cognitively and emotionally during role performances”. Kahn (1990) operationalized work engagement as the physical involvement in tasks, cognitive attention and emotional connection to others when performing tasks. Louis and Smith (1992) pointed out that “in primary or secondary education, teacher engagement refers to a teacher's psychological investment in an effort toward teaching the knowledge, skills, and crafts he or she wishes students to master” (p. 120). Raina and Khatri (2015) stated that some factors, such as educational experience, learners' aptitude, class size, school location, class the school, classroom contexts, classroom management, task management, novelties in educational contexts and instruction, feedback received by learners and principal, interaction with colleagues, and opportunities for cooperation with others are critical in teacher engagement. Timms and Brough (2013) emphasized the importance of two theoretical models for teachers' work engagement, namely, job-demands-resources model (Demerouti et al., 2001) and the self-determination model (Ryan and Deci, 2000). They asserted that the job-demands-resources model, compared to the self-determination one, is widely used in educational contexts, since it is applied to explain teacher burnout along with job involvement.

Teacher engagement has been investigated in many studies, which considered its relationship with their demographical variables. Topchyan and Woehler (2021) found that full-time female educators with higher levels of social involvement with learners have more degrees of work engagement and job satisfaction. They also found a significant correlation between work experience and work engagement. In a study on the validity of the Utrecht Work Engagement Scale, Klassen et al. (2012) found the modest effect of gender, age, and, and years of experience on the involvement of teachers in academic contexts.

Benesch (2018) stated that language educators' feelings can be considered as the causes of work engagement. Likewise, Ghanizadeh and Moafian (2010) showed that positive affectivity, including enjoyment and hope are critical in shaping educators' engagement. Consequently, the prominence of controlling positive and negative feelings is remarkable in educational contexts where feelings play an important role in adjusting the quality of instruction and engagement. Jennings and Greenberg (2009) mentioned that instructors with high social and affective capabilities could positively discover applied solutions in challenging contexts and build up their engagement. Teacher efficacy, as a construct of positive psychology, has been examined by Perera et al. (2018). They found that resiliency mediates the relationship between teachers' self-efficacy and work engagement and emotional engagement. In another study on Korean teachers, the findings of Song et al. (2018) revealed that teacher self-efficacy is significantly correlated with work engagement. Zeng et al. (2019), in their study in the Chinese context, demonstrated that teachers' growth mindset, wellbeing, and resilience strongly predict job engagement. They also found out that wellbeing and grit mediate the correlation between work engagement and a growth mindset. Diener et al. (2020) mentioned that positive feelings affect teachers' performance in language teaching together with long-term work involvement, positive attitudes, resourcefulness, operative teaching strategies, and teacher-learner rapport. They argued that positive feelings activate upward spirals, since the positive results predict upcoming rises in positive feelings, and result in work engagement and wellbeing. Greenier et al. (2021) showed that teacher wellbeing and emotional regulation strategies significantly correlate with teacher engagement. They argued emotional regulation strategies used by teachers are effective for their involvement in doing educational tasks. Sonnentag et al. (2008) also found a negative and significant correlation between work engagement and emotional exhaustion. Han et al. (2020) also listed the main reasons for teachers' less work engagement and exhaustion: teaching difficulties, teaching-research conflict and new challenges in the teacher-learner relationship.

### The Relationship Between Work Engagement and Ambiguity Tolerance

Few investigations have been done on the relationship between engagement and ambiguity tolerance among learners. Yu et al. (2021) confirmed the positive role of learners' ambiguity tolerance and resilience in their academic engagement. They mentioned that when learners cope with new situation and information in educational contexts, they may not respond appropriately to the new contexts which result in stress. Therefore, they asserted that learners with high levels of ambiguity tolerance can engage more in classroom contexts. Furthermore, they mentioned that EFL teachers should be responsible for catering more student-friendly and less anxiety-inducing educational contexts setting, which encourages learners to be more involved in academic contexts. Mirsadegh et al. (2021) found the mediating role of academic resilience in the correlation between learners' ambiguity tolerance and academic engagement. They argued that when students are cognizant of their educational environment, the techniques used by teachers, and the upcoming instructive plans, they try to have higher levels of commitment to be more engaged in educational contexts. They maintained that ambiguity tolerance enables them to admit the individuals' assertions that are contrary to their principles and beliefs. Moreover, they mentioned that continuing inspiration of learners by their families can motivate them to be tolerant about ambiguous contexts, and to engage more in educational contexts.

However, the relationship between teacher engagement and tolerance of ambiguity is not widely studied in the literature. In a study in relation to teachers' exhaustion and burnout in educational contexts, Fisherman (2015) believed that ambiguity in educational contexts can explain the relationship between teacher identity development and teacher burnout. He found out that kindergarten teachers, compared to elementary and high school teachers, were more engaged in the educational contexts, and they have lower levels of teacher burnout. They argued that Kindergarten teachers had a comparatively well-defined set of expectations, and the educational conditions were less ambiguous to them. They felt accountable to their parents and supervisors; therefore, they did their best in order to meet their requirements. The study conducted by Mérida-López et al. (2017), revealed that ambiguity and conflict are negatively correlated with educators' vigor and dedication as two components of teachers' work engagement. Their study showed the interaction of ambiguity, conflict, emotional intelligence in predicting work engagement. Rezaei et al. (2019), in their study, found that experienced teachers, compared to novice ones, tend to be more tolerant in ambiguous situations. They asserted that novice teachers with lower levels of ambiguity tolerance are less involved in educational contexts.

### The Relationship Between Ambiguity Tolerance and Emotional Intelligence

Some studies have been done on learners “emotional intelligence and their tolerance of ambiguity. Using Ely's (1989) Ambiguity Tolerance Scale, and Schutte et al.'s (1998) Emotional Intelligence Scale, Rastegar and Mehrabi Kermani (2015) found that learners” emotional intelligence is not significantly correlated with the tolerance of ambiguity. However, they argued that “putting into our mind the special features and capabilities of this intelligent use of emotions in dealing with uncertainties and problems facing a learner especially in new context makes it impossible to ignore its role” (p. 8). In the same vein, Nosratinia et al. (2013) showed a non-significant relationship between learners' emotional intelligence and ambiguity tolerance. Vahedi and Fatemi (2016) demonstrated a non-significant correlation between emotional intelligence and tolerance of ambiguity. However, these constructs are positively correlated with learners' willingness to communicate. On the other hand, Pavlova and Kornilova (2013) found out that creativity and am as predictors of ambiguity tolerance significantly predict learners; emotional intelligence in decision-making. However, little is known about the relationship between emotional intelligence and the tolerance of ambiguity among teachers.

### The Relationship Between Emotional Intelligence and Work Engagement

Research has found that emotional intelligence is related to concepts similar to engagement such as job satisfaction (Perera et al., 2018; Chan et al., 2020) work attitudes, behavior, and outcomes (Carmeli, 2003), and self-esteem (Lavy and Naama-Ghanayim, 2020). Puertas Molero et al. (2019) suggested that due to the strong relationship between emotional intelligence and several psychological well-being components, there is potential with regard to emotional intelligence predicting engagement in the workplace. Emotional intelligence involves an awareness and regulation component, which is important in maintaining positive emotional states (Herman, 2012). Dewaele et al. (2018) found emotional intelligence to be related positively to important employment experiences and individuals' emotional attachment to their current careers and jobs. Moreover, Inceoglu and Warr (2011) found that engaged individuals were more likely to be emotionally stable, socially proactive and achievement oriented. Lamberti's (2010) study identified engagement and emotional intelligence as two of six drivers of organizational energy.

Some studies have been done on the relationship between learners' emotional intelligence and academic engagement. Martín et al. (2021) found a significant correlation between three components of engagement, including vigor, dedication and absorption with secondary school learners' emotional intelligence and self-esteem. Besides, they found that self-esteem mediates the correlation between emotional intelligence and engagement. They argued that learners' emotional intelligence develops optimism toward learning and educational contexts. They mentioned that learners who are able to properly control their feelings have the motivation to engage enthusiastically in academic contexts. Zhoc et al. (2020) also investigated university students' emotional intelligence and all types of engagement. Their study revealed that emotional intelligence is significantly correlated with learners' social engagement, emotional engagement, and cognitive engagement. They mentioned that “emotional intelligence develops both academic and social functioning that enhances student engagement” (p.15). Maguire et al. (2016) controlled the effect of demographical features, such as gender and age on learner engagement. They found that emotional intelligence is significantly correlated with learners' cognitive and affective engagement.

Concerning teachers' work engagement, Mérida-López et al. (2017) investigated the influence of emotional intelligence and stress on teachers' work engagement. They argued that the development of emotional intelligence can reduce stress arising from ambiguous information within instructional environments, which, in turn, enhances teacher engagement. They justified their results using the conservation of resources theory, which postulates that “a context with high demands can lead to the particular salience of resources, which therefore strongly influence engagement” (p.10). They mentioned that teachers tend to employ their emotional processing resources when coping with stress associated with unclear responsibilities, chores, and tasks. Abiodullah et al. (2020) approved the significant correlation between teacher engagement and emotional intelligence. They argued that the teacher-learner rapport and the relationship between teachers and colleagues result in work engagement. D'Amico et al. (2020) found out that Italian teachers' emotional intelligence is positively correlated with work engagement and job satisfaction. They argued that emotionally intelligent teachers are enthusiastic and involved with their job when encountering numerous stressors. Sudibjo and Sutarji (2020), in their study, found that job satisfaction, wellbeing, and emotional intelligence are positively correlated with educator' work engagement. They argued that a contented, emotionally intelligent employee with a higher level of wellbeing tends to be more productive and engaged in an organization. The study by Butakor et al. (2021) also revealed that job satisfaction is an important factor in the correlation between teachers' emotional intelligence and work engagement. They argued that educators with high levels of emotional intelligence are inclined to be satisfied with their job and this satisfaction is transformed into teacher involvement in academic contexts.

The study conducted by Mérida-López et al. (2019) revealed that teachers' emotional intelligence can boost the correlation between self-appraised stress and job involvement. They used Bakker and Demerouti's (2017) JD-R theory, and Cote's (2014) emotional intelligence model in order to explain the buffering effect of emotional intelligence in the relationship between self-appraised stress and teachers' work engagement. They argued that emotional intelligence plays the role of personal resource, contributing teachers to cope with the harmful influences that self-appraised stress has on work engagement, but it does not diminish the impacts of affective strains on teachers. In the same vein, Pena et al. (2012) found out that elementary and primary educator's emotional intelligence, work engagement, perceived stress, and life satisfaction are correlated with each other. They mentioned that emotionally intelligent teachers have lower levels of stress, which, in turn, leads to an increase in work engagement and life satisfaction.

## Methodology

### Participants

The current study involved 364 Chinese EFL teachers, and only 322 of them were valid cases. Among them, there were 96 male teachers and 226 female teachers, aged 25 to 52, whose majors included Business English, Teaching English to Speakers of Other Languages (TESOL), English Literature and English Translation Studies. It is worth noting that the majority of the participants held their Master's degree (87%) and the minority of them were Ph.D. holders (13%). The participants were recruited from the following five provinces, namely Shandong, Heilongjiang, Hunan, Hubei, and Sichuan.

### Instruments

Three types of questionnaires, including Schutte's Self Report Emotional Intelligence Test (SSEIT), Multiple Stimulus Types Ambiguity Tolerance Scale-II (MSTAT-II), and Self-report Engagement Questionnaire were used in this study. The Schutte's Self-Report Emotional Intelligence (SSREI) scale by Schutte et al. (1998) is comprised of 33, 5-point Likert scale items, three of which are negatively keyed. Previous investigations have found the total scores on the SSREI to be acceptably internally consistent (e.g., 0.90; Schutte et al., 1998). MSTAT-II is designed and validated by McLain (2009) to measure individuals' general tolerance/intolerance for ambiguity. The scale consists of 13 items scored on a 5-point continuous Likert scale ranging from 1 (strongly disagree) to 5 (strongly agree). However, for items # 1, 2, 3, 4, 5, 6, 9, 11, and 12, which are negatively worded, scoring should be reversed. Individuals' low scores represent their aversion to ambiguity, whereas their high scores represent their interest in ambiguity. McLain (2009) reported a Cronbach's alpha reliability coefficient of 0.82 for the scale. In this study, the scale's estimated Cronbach's alpha reliability coefficient was equal to.93. Teachers' work engagement was assessed with 24 self-constructed items that were formulated in English. The engagement items are supposed to reflect three underlying dimensions: *Vigor* (VI) (9 items; e.g., “When I get up in the morning, I feel like going to class/work”); *Dedication* (DE) (8 items; e.g., “I'm enthusiastic about my study/job”), and *Absorption* (AB) (7 items; e.g., “When I'm studying/working, I forget everything around me”).

### Procedure

Before collecting the data, we translated the English validated questionnaires including SSEIT, MSTAT-II, and Self-report engagement Questionnaire by using the backward translation. In the meantime, to ensure the reliability and validity of our data collection, the consults of some experts in this area, who have published their academic papers in some prestigious SSCI-indexed journals are used in this study. This survey was administered online through Wenjuanxing, a popular data collection tool in China and the participants were provided the consent to be willing to participate in the present study and also were informed that they were able to withdraw their data without offering any reasons. The data collection lasted for around one and half months and finally we got 322 valid participants that can be used for further statistical analysis.

## Results

To make sure of the reliability of the questionnaires administered in this study, three Cronbach alpha tests were run.

Table 1 shows that work engagement questionnaire (0.80), emotional intelligence questionnaire (0.95), and ambiguity tolerance questionnaire (0.97) had satisfactory reliability indices. To decide upon the parametric or non-parametric analysis, a test of normality was run. The results are shown in the following.

**Table 1:** Reliability of the instruments.

**Questionnaire**	**Cronbach's Alpha**	**N of Items**
Work engagement	0.80	33
Emotional intelligence	0.95	17
Ambiguity tolerance	0.97	13

The indices of Kolmogorov-Smirnov (Table 2) show that the distribution of data was not normal for any of the variables since p value is lower than the significance level (*p* < 0.05). Consequently, the non-parametric analysis, Spearman Rho test, was used.

**Table 2:** Test of normality.

	**Kolmogorov-Smirnov** ^**a**^			**Shapiro-Wilk**		
	**Statistic**	**df**	**Sig**.	**Statistic**	**df**	**Sig**.
WE	0.199	322	0.000	0.901	322	0.000
EI	0.065	322	0.002	0.941	322	0.000
AT	0.176	322	0.000	0.789	322	0.000

^a^*Lilliefors significance correction*.

### The First Research Question

The first research question deals with the relationship among three variables of this study (i.e., work engagement, emotional intelligence and ambiguity tolerance) which was calculated through running a Spearman Rho correlation test. Table 3 shows the relationship among the Iranian EFL learners' work engagement, emotional intelligence and ambiguity tolerance.

**Table 3:** Correlations among work engagement, emotional intelligence, and ambiguity tolerance.

			**WE**	**EI**	**AT**
Spearman's rho	WE	Correlation coefficient	1.000	0.658**	0.622**
	EI	Correlation coefficient	0.658**	1.000	0.679**
	AT	Correlation coefficient	0.622**	0.679**	1.000
		Sig. (2-tailed)	0.000	0.000	0.000
		N	322	322	322

**Correlation is significant at the 0.01 level (2-tailed).

**Figure 1:**
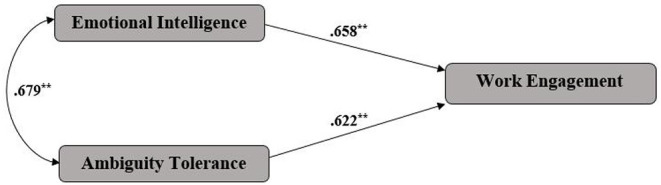
Model of relationships between emotional intelligence, ambiguity tolerance, and work engagement. **Significant level (0.0 > 0.05).

As Figure 1 demonstrates, there are positive (0.658, 0.652) and significant (sig = 0.000) relationships among work engagement, emotional intelligence and their ambiguity tolerance. It can be concluded that if learners' indices of emotional intelligence and ambiguity tolerance increase, the index of learners' work engagement decreases.

### The Second Research Question

The second research question deals with measuring the predictability power emotional intelligence and ambiguity tolerance for work engagement. To this end, a linear multiple regression analysis was performed in the following tables.

The model summary Table 4 shows that how much of the variance in the dependent variable [scores obtained from the dependent variable (work engagement)] can be explained by the model (which included the variables of emotional intelligence and ambiguity tolerance). In this case, the value was 0.71 (R^2^ = 0.509). Expressed as a percentage, it implies that the model explained 50.9 percent of the variance in scores from work engagement.

**Table 4:** Model summary for work engagement, emotional intelligence and ambiguity tolerance.

**Model**	**R**	**R square**	**Adjusted R square**	**Std. error of the estimate**
1	0.714^a^	0.509	0.506	11.180

^a^*Predictors: (Constant), AT, EI*.

To assess the statistical significance of the results, Table 5 analyzed that whether or not the model (which includes AT and EI) is a significant predictor of the work engagement. This tested the hypothesis that multiple R in the population equals zero (0). Thus, the model reached statistical significance [F = (2, 319) = 165.65, Sig = 0.000, this really means *p* < 0.05].

**Table 5:** ANOVA for work engagement, emotional intelligence, and ambiguity tolerance.

**Model**		**Sum of squares**	**df**	**Mean square**	**F**	**Sig**.
1	Regression	41412.94	2	20706.47	165.65	0.000^b^
	Residual	39874.94	319	125.00		
	Total	81287.89	321			

^a^*Dependent Variable: WE*.

^b^*Predictors: (Constant), AT, EI*.

Tests to see if the data (Table 6) met the assumption of collinearity indicated that multicollinearity was not a concern (EI Scores, Tolerance = 0.86, VIF = 1.15; AT, Tolerance = 0.86, VIF = 1.15).

**Table 6:** Collinearity statistics.

**Model**		**Collinearity statistics**	
		**Tolerance**	**VIF**
1	EI	0.86	1.15
	AT	0.86	1.15

In this study, the researchers were interested in *comparing* the contribution of each independent variable; therefore, they used the beta values. Looking down the Beta column (Table 7), they found that the largest beta coefficient was 0.61 (sig = 0.000), which was for emotional intelligence. This means that this variable made the strongest contribution to explaining the dependent variable, when the variance explained by all other variables in the model was controlled. The Beta value (0.20) for the other variable (i.e., ambiguity tolerance) was also significant (sig = 0.000).

**Table 7:** Coefficients for work engagement, emotional intelligence, and ambiguity tolerance.

**Model**		**Unstandardized coefficients**		**Standardized coefficients**	**t**	**Sig**.
		**B**	**Std. error**	**Beta**		
1	(Constant)	−119.96	11.57		−10.36	0.000
	EI	1.026	0.071	0.611	14.50	0.000
	AT	1.153	0.232	0.209	4.96	0.000

^a^*Dependent Variable: WE*.

## Conclusion

This study aimed to investigate the relationship between Chinese teachers' work engagement, emotional intelligence, and ambiguity tolerance. It is revealed that teachers' work engagement, ambiguity tolerance and emotional intelligence are significantly correlated with each other. Moreover, our findings showed that teachers' emotional intelligence and ambiguity tolerance significantly predict work engagement. Our findings hint that emotionally intelligent instructors with higher levels of ambiguity tolerance are more engaged in their instruction and the provision of innovative methodologies for learners.

## Discussion

The findings of this study can be corroborated by Mérida-López et al. (2017) who examined educators' emotional intelligence, and its effect on teachers' work engagement. It can be argued that teachers' emotional intelligence, which develops teacher-learner rapport can reduce job stress, which, in turn, fosters teachers' involvement in academic contexts. Moreover, the findings of this study are consistent with Butakor et al.'s (2021) study, wherein teachers' emotional intelligence and academic engagement are indirectly correlated with each other, and job satisfaction acts as a mediator in this correlation. Overall, the finding of this study is in accordance with findings reported by Sudibjo and Sutarji (2020), who found a positive correlation between teachers' emotional intelligence and work engagement. We can also suggest that the emotional construct, such as wellbeing, can mediate the correlation between job involvement and emotional intelligence.

From emotional intelligence theory, personal resources such as emotional intelligence may serve a moderating function through direct effects on the way individuals appraise and deal with a threatening situation or by implementing changes in problem-solving behaviors. Therefore, these emotional resources may lead individuals to handle threats more constructively and thus experience reactions that are more positive at work. Furthermore, emotional intelligence is considered an antecedent of work engagement (Bakker et al., 2014). Consistent with the JD-R model, social and personal resources such as emotional intelligence would influence work engagement. Accordingly, personal resources such as emotional intelligence might energize teachers, encourage their persistence, and make them focus on their efforts. In other words, these emotional resources might foster engagement in terms of vigor (energy), dedication (persistence), and absorption (focus) (Extremera et al., 2012).

Another finding is that emotionally intelligent teachers can tolerate the ambiguities produced by educational contexts. However, when comparing our results to those of Nosratinia et al. (2013), Rastegar and Mehrabi Kermani (2015), and Vahedi and Fatemi (2016), which showed a non-significant relationship between these two constructs among learners, it must be pointed out that the results of this study show a positive relationship between emotional intelligence and ambiguity tolerance. A similar pattern of results was obtained in Rezaei et al. (2019) who showed that teachers with higher levels of ambiguity tend to engage more in academic contexts.

This study includes some pedagogical implications for teacher educators, policy-makers, and advisors. To improve teachers' work engagement, teacher educators and mentors can emphasize instructors attach importance to the constant academic development and critical thinking to enhance their instructional method. Instructors should be directed to be well-informed about instructive issues and take advantage of improved learning chances. It is also suggested that teacher educators highlight interaction tools, like mobile applications, which encourage teachers and learners to interact and scaffold that increase efficacy. They should develop confidence and competence among in-service teachers to entice learners' interests and engage them in the learning process. Teacher educators can enquire about syllabus, education, and schedules to engage them in thinking about educational conditions. They should pay attention to their syllabus designs, and include their opinions in their decision-making about syllabus designs. They can also prove a context for teachers to engage in learner-centered projects. Teacher educators can also decrease teachers' ambiguity by providing a situation in which teachers can observe the instruction of their peers. They can also provide scaffolding among teachers to remove the unclear issues. Teacher educators can also discuss theories of language learning and teaching methodologies, and they can ask the opinions of teachers to remove the barriers in educational contexts and clarify teaching and learning issues. Finally, teacher educators can improve teachers' emotional intelligence. They can hold workshops and provide some strategies to improve teachers' emotional intelligence. They can also emphasize modeling teacher-student rapport, taking action and improving listening, and trying not to interrupt learners while they are speaking. They can give some instructions to use gestures in communication with learners. Moreover, some recommendations, such as using non-monotonous speech, smiling during speech, looking at the whole class during talking, and having a relaxed posture can be presented in the workshops.

Educational policy-makers should hire experienced teachers, as the instructive experience can be an important issue for increasing engagement, emotional intelligence, and ambiguity tolerance among teachers. Educational policy-makers can increase teacher engagement by holding academic workshops that offer teachers some authentic activities. They can ask teachers to do their best within varied educational contexts. Educational policy-makers must build up teaching effectiveness by providing contexts for observations of other teachers' activities and mastery experiences to decrease teacher ambiguity. Policy-makers should also provide critical thinking, creativeness, and motivation to the education in classrooms, which encourages work engagement. The importance of engagement and ambiguity tolerance can motivate advisors to expand their horizons to identify teachers' sources of engagement and ambiguity to remove their barriers.

This study has some limitations. Most of the participants of this study are from five provinces and few from other cities. This can cause a generalization issue. Next, the number of participants in studies using this quantitative approach is often limited. A small number of teachers participated in this research. Beliefs and cognitions held by this sample of participants may not inevitably depict the cognitions of a larger population.

Future studies should aim to replicate results in larger contexts. In future work, investigating teachers' ambiguity tolerance, emotional intelligence and their role in work engagement in technology-supported contexts, numerous cultural backgrounds, and among teachers with different educational experiences can be important for future studies. Some investigations need to be done on the effect of teachers' ambiguity tolerance on their motivation in traditional and virtual contexts. Furthermore, the relationship between teacher proficiency level of foreign language, and its effect on their work engagement and ambiguity tolerance should be considered in the future. Furthermore, case and phenomenological investigations, which provide us with the reasons behind teachers' work engagement and emotional intelligence are required to be done. Some investigations should be done on the relationship between positive psychological constructs such as enjoyment, grit, positive affectivity, resilience, and teachers' ambiguity tolerance. In addition, future research should examine the roles of negative factors such as anger, frustration in ambiguity tolerance, and work engagement.

## Data Availability Statement

The original contributions presented in the study are included in the article/supplementary material, further inquiries can be directed to the corresponding author.

## Ethics Statement

The studies involving human participants were reviewed and approved by Shandong Management University Academic Ethics Committee. The patients/participants provided their written informed consent to participate in this study.

## Author Contributions

The author confirms being the sole contributor of this work and has approved it for publication.

## Conflict of Interest

The author declares that the research was conducted in the absence of any commercial or financial relationships that could be construed as a potential conflict of interest.

## Publisher's Note

All claims expressed in this article are solely those of the authors and do not necessarily represent those of their affiliated organizations, or those of the publisher, the editors and the reviewers. Any product that may be evaluated in this article, or claim that may be made by its manufacturer, is not guaranteed or endorsed by the publisher.
